# Development and characterization of niosomal gel of fusidic acid: in-vitro and ex-vivo approaches

**DOI:** 10.1080/15685551.2022.2086411

**Published:** 2022-06-09

**Authors:** Muhammad Khurram Waqas, Haleema Sadia, Muhammad Imran Khan, Muhammad Ovais Omer, Muhammad Irfan Siddique, Shaista Qamar, Muhammad Zaman, Muhammad Hammad Butt, Mian Waqar Mustafa, Naeem Rasool

**Affiliations:** a Institute of Pharmaceutical Sciences, University of Veterinary and Animal Sciences, Lahore, Pakistan; b Riphah Institute of Pharmaceutical Sciences, Riphah University Lahore Campus, Pakistan; c Department of Pharmacology and Toxicology, University of Veterinary and Animal Sciences Lahore, Pakistan; d Faculty of Pharmacy, University of Central Punjab, Lahore, Pakistan; e Department of Pharmacy, Forman Christian College University, Lahore, Pakistan

**Keywords:** Fusidic acid, niosomes, topical, Carbopol gel, permeation

## Abstract

Niosomes are multilamellar vesicles that efficiently deliver active substance into skin systemic circulation or skin layers. They are used in topical drug delivery system to enhance the skin permeation of active substance. So, the prime objective of this study was to develop a niosomal gel of fusidic acid to increase its skin permeation. Different formulations of niosomes of fusidic acid were designed by varying the cholesterol to surfactant ratio. Formulations containing fusidic acid, cholesterol, dihexadecyl pyridinium chloride, Span 60, or Tween 60 were prepared by thin film hydration method in rotary evaporator. The thin film formed in rotary flask was hydrated by phosphate buffer saline of pH 7.2. The niosomes formed were characterized through entrapment efficiency, size, polydispersity index (PDI), and zeta potential. The S3 formulation containing span 60 showed the highest entrapment efficiency (EE) of niosomes, so it was incorporated into Carbopol gel. Determination of pH, spreadability, rheological, and *ex vivo* permeation studies was conducted of niosomal gel. The results of *ex vivo* permeation studies showed high permeation of fusidic acid when gel was applied to an albino rat skin. According to the results and previous studies of niosomes, it can be concluded that niosomes enhanced the permeation of fusidic acid through the skin.

## 1. Introduction

Fusidic acid is bacteriostatic agent obtained from the fungus *Fusidium coccineum* in 1962. It belongs from class fusidanes [1]. It is a steroid antibiotic of narrow spectrum, which is predominantly active against gram-positive bacteria. It is mainly active against *Staphylococcus aureus, S. epidermis, Clostridium spp*., and corynebacterial [2]. *S. aureus* is one of the species that is a leading threat to public health and causes morbidity or mortality [3,4]. Fusidic acid inhibits protein synthesis of bacteria by interfering with its elongation factor G (translocase) and may be by other mechanisms. Fusidic acid acts through four phases, i.e., Initiation, elongation, translocation, and release. All four phases are engaged in protein synthesis and are actuated by four proteins (IF-2, Initiation factor-2; EF-Tu, elongation factor thermo unstable; EF-G, elongation factor-G; and RRF, ribosome recycling factor). Moreover, all four proteins have GTPase activity, and if any protein gets repressed, then automatically it blocks protein synthesis [5]. Using quench flow and stopped flow experiments in a biochemical system for protein synthesis and taking advantage of separate time scales for inhibited (10 s) and uninhibited (100 ms) elongation cycles, a detailed kinetic model of FA action was obtained. FA targets EF-G at an early stage in the translocation process (I), which proceeds unhindered by the presence of the drug to a later stage (II), where the ribosome stalls. Stalling may also occur at a third stage of translocation (III), just before release of EF-G from the post-translocation ribosome. We show that FA is a strong elongation inhibitor [6]. It is bacteriostatic, but at high concentration, it acts as bactericidal [2]. Fusidic acid is mainly used in skin and soft tissue infections. The common skin infections in which fusidic acid is used are impetigo, erythrasma, bullous impetigo, psoriasis, folliculitis, furuncles, carbuncles, contagiosa, infected wounds, and burns [7]. In a research investigation by Ulkur et al. on MRSA-infected full-thickness surface of experimental animal burn wounds, MRSA was eliminated in burn eschars that were managed topically by 2% fusidic acid formulation, whereas silver dressing and chlorhexidine acetate therapy attained only minor decline in the bacterial load [8]. Vingsbo et al. noticed considerable log reductions of MRSA, 2.9 log following 3 days, and 4.2 log following 6 days, in experimental skin infections with fusidic acid topical delivery [9]. Vanangamudi et al. patented copious topical creams containing fusidic acid that can be used on bacterial contagion skin surface, together with burn wound infections [5]. Furthermore, the patented topical creams may also contain an anti-inflammatory steroidal drug in addition to chitosan as a biopolymer and an antifungal modality for augmenting antimicrobial activity.

The oral and parenteral administration of fusidic acid poses adverse effects such as phlebitis, rhabdomyolysis, hepatotoxicity, diarrhea, and gastrointestinal discomfort. These adverse events occur because of the wide systemic non-specific distribution of fusidic acid, which decreases its therapeutic efficacy at the site of action, i.e skin [10]. For topical dosage form, the penetration of fusidic acid through the normal skin is low. Its penetration is also enhanced in skin diseases such as dermatitis warts or seborrhea [11].

The topical route for drug administration has many benefits over other routes of administration. It has lesser side effects and better patient compliance. Topical route of drug administration is a good alternative to needle injection or oral intake of medications [12]. Topical administration offers advantages such as avoidance of first pass effect, self-administration with ease, convenience, generally good acceptance by patients, and reduced toxicity due to minimum plasma drug levels [13]. Although this route has benefits, but still poor skin permeability of many drugs makes its use limited. To overcome this problem, many formulation approaches have been attempted such as iontophoresis, electroporation and nanovesicular drug delivery systems, and vesicular drug delivery systems [14]. Vesicular systems are now widely used to overcome this barrier in topical drug delivery system. These vesicular systems consist mainly of liposomes and niosomes.

Recently, owing to advances in drug formulation strategies, niosomes have attained significant attention by pharmaceutical scientists and being investigated as alternate carriers to that of liposomes. Niosomes or commonly known as non-ionic surfactants are used widely as an alternate carrier to liposomes. They have comparable physical properties. Niosomes enhance drug permeation into skin by distorting membrane properties of stratum corneum, and it fuses with upper layer of skin. They also showed drug deposition in different skin layers [15].

Niosomes when used in topical drug delivery system act as penetration enhancers, serve as local storehouse for sustained release of drug, increase solubility of poorly soluble drugs, and act as rate-limiting membrane barrier for controlled delivery systems. Different niosome–skin interaction mechanisms have been proposed in literature for niosomes such as diffusion through skin, interlinkage with skin lipids and alteration of structure of stratum corneum, thereby increasing skin permeation [16]. The surfactants as essential component of niosomes structure act as permeation enhancers and direct fusion of vesicles with stratum corneum [17] . Simultaneously, they serve as a local depot for the sustained and controlled release of dermally applied bioactive moiety [18]. Niosomes are relatively more stable and economical, which render it preferable approach compared to liposomes. Therefore, the application of niosomes is predicted to improve the penetration of fusidic acid through skin. Previously, a number of nanosystems have been explored in the past for the treatment of wounds and associated infections including nanoemulsions, solid lipid nanoparticles, liposomes, ethosomes, niosomes, polymeric nanoparticles, dendrimers, micellar systems, and carbon-based nanostructures [19,20]. Fusidic acid (FA)-based topical liposomal gel was formulated by Wadhwa et al. to treat the bacterial infection concomitantly occurring in the condition of plaque psoriasis. The FA–liposomal gel formulation was found to be superior in terms of skin permeation and retention studies carried for 24 h. Around 75 ± 1.2% permeation was observed for FA–liposomal gel, 53 ± 2.5% for commercial cream (Fucidin™), and 59 ± 1.7% for FA hydrogel. Significant results were exhibited by FA–liposomal gel in psoriasis-induced mouse tail model as compared to commercial and hydrogel groups [21]. In a study conducted by Thakur et al., chitosan-lipid based nanoparticles loaded with fusidic acid were developed and evaluated. The developed nanocarriers offered nanometric size (284.67 ± 5.67 nm), sustained drug release (79.31 ± 0.45%), and enhanced drug permeation (72.09 ± 1.26%). The changes in viability of HaCat cells were insignificant, indicating the safety profile of nanocarriers. The administration of nanocarriers loaded with fusidic acid demonstrated 5-times and 4-times decrease in its inhibitory concentration against MRSA 33591 and MSSA 25921, respectively, along with antibacterial activity for a longer duration [22] . However, there is limited information available for niosomal delivery of fusidic acid. Therefore, the current research study intends to explore the potential of niosomes for effective dermal delivery of fusidic acid to manage skin infections. Sorbitan monostearate (span 60) and polyoxyethylene sorbitan monostearate (tween 60)-based fusidic acid niosomes were prepared using thin film hydration method. Different surfactant to cholesterol ratios were investigated to optimize the niosomes. The developed niosomes were characterized for entrapment efficiency, vesicle size, polydispersity index (PDI), and permeation potential of niosomes.

## 2. Materials and methods

### 2.1. Materials

Fusidic acid micro powder (Joyang laboratories) was gifted by Valor Pharmaceuticals Pvt Limited, Islamabad. Cholesterol was purchased from DAEJUNG Chemicals and Metals co., Korea. Sorbitan monostearate (span 60) and polyoxyethylene sorbitan monostearate (tween 60), chloroform, dihexadecyl phosphate chloride, and Carbopol 934 were purchased from Merck, Germany. Triethanolamine was procured from Sigma Aldrich (St Louis, MO, USA). Distilled water was locally prepared in the Institute of Pharmaceutical Sciences, UVAS, Lahore, Pakistan. All chemicals were of analytical grade.

### 2.2. Preparation of fusidic acidloaded niosomes by thin film/hand shaking method

Different niosomal formulations of fusidic acid were prepared by varying the surfactant to cholesterol ratios (5:5, 7:3, and 6:4). Niosomes were prepared using traditional thin film hydration method with slight modification reported elsewhere [23]. Two sets of formulations (each set of 3) were prepared using Span 60 (S_1_, S_2_, S_3_) and Tween 60 (T_1_, T_2_ and T_3_) as surfactants with different molar ratios of cholesterol. Briefly, 400 µmole of surfactant/cholesterol in different molar ratios were dissolved in 10 mL of chloroform at ambient temperature, followed by further addition of 20 mg of active fusidic acid and small amount (4.25 mg) of dihexadecyl pyridinium chloride as charge-inducing agents (Table 1). The chloroform was removed at 66°C, under reduced pressure, using rotary evaporator (Heidolph Hei VAP Rotary Evaporator, Germany). The chloroform removal was accomplished at 120 rpm for 1 hour until formation of lipidic thin film. The flask was placed in desiccator overnight to remove any trace of organic residue. The complete dried film was hydrated in 10 ml phosphate buffer saline (pH 7.2) by hand shaking flask for half an hour. The aqueous niosomal formulations were kept aside to get mature and stored in refrigerator at 4°C for further characterization.

**Table 1.** Composition of niosomes loaded with fusidic acid

Formulation codes		Surf: CHO	Fusidic acid (mg)	Cholesterol		Hexadecyl pyridinium chloride (mg)	Span 60		Tween 60	
				µ. mol	mg		µ. mol	mg	µ. mol	Mg
Span 60	NS1	5:5	20	200	96.5	4.25	200	107.75	-	-
	NS2	6:4	20	160	77.2	4.25	240	129.3	-	-
	NS3	7:3	20	120	57.9	4.25	280	150.85	-	-
Tween 60	NT1	5:5	20	200	96.5	4.25	-	-	200	327.9
	NT2	6:4	20	160	77.2	4.25	-	-	240	393.5
	NT3	7:3	20	120	57.9	4.25	-	-	280	459.0

NS = Niosomes based on Span 60

NT = Niosomes based on Tween 60

### 2.3. Preparation of niosomes-based gel

According to the results of characterization of fusidic-acid loaded niosomes, niosomes coded as NS3 was chosen to be integrated into gel dosage form. In first step, plain gel was prepared by incorporating 1 g Carbopol 934 in 100 mL distilled water at 50°C through a continuous stirring at 450 rpm using homogenizer (Euro star, IKA-D-230 Germany). Methyl paraben and propyl paraben were added in small quantities (0.02%) as preservatives to the gel. The NS3 formulation equivalent to 20 mg of fusidic acid was mixed thoroughly with the above mentioned Carbopol gel. Then it was allowed to swell for 24 hours. Finally, weighed quantity of triethanolamine was added as a neutralizer to increase the pH to 6.4 of the prepared Carbopol 940 mixture, and formation of gel occurred [24].

### 2.4. Evaluation of niosomes

#### 2.4.1. Fourier transform infrared spectroscopy (FTIR)

FTIR was performed to assess possible physical interaction between fusidic acid, Span 60, Tween 60, cholesterol, and hexadecyl pridinium chloride. Spectra of individual niosomal components and formulations were measured using a pike single-bounce attenuated total reflectance (ATR) cell equipped with a ZnSe single crystal (Bruker, Tensor 27 series, Germany). For solid samples (pure drug and cholesterol), the samples were added to the ATR cell and measured directly. For liquids (niosomal formulation), the samples were placed directly on the small crystal spot, and lever having concave surface is placed over it in order to prevent evaporation. Spectral scanning was taken in the wavelength region between 4000 and 600 cm^−1^ at a resolution of 4 cm^−1^ with scan speed of 2 mm/s.

#### 2.4.2. Percent drug entrapment efficiency (%EE) studies

The entrapment efficiency of fusidic acid in niosomes was determined using cooling centrifuge (Sigma 1–14, Germany) described previously [22]. The niosomal formulations were subjected to centrifugation at 12,000 rpm for 30 mins at 4°C. The supernatant was decanted, and free drug contents were analyzed by UV/visible spectrophotometer (Uvikon XL, Bio-Tec Instruments, Bad Friedrichshall, Germany) at *λ*max of 210 nm. The %EE was then calculated by using following formula:

(1) $$\%Drug\;entrapment=\frac{Total\;drug-Drug\;in\;supernatant}{Total\;drug}x100$$

#### 2.4.3. Vesicle size, polydispersity index (PDI), and zeta potential

Vesicle size, PDI, and zeta potential of niosomes were determined by dynamic light scattering (DLS) experiments (Zetasizer Nano ZS, Malvern instruments, England). The samples were diluted 100 times with double distilled water prior to analysis. The samples were placed in cuvette of Zetasizer, and the data was recorded. The results were recorded as an average of three measurements.

#### 2.4.4. Morphological evaluation

The morphology of fusidic acid-loaded niosomes was evaluated using scanning electron microscope (SEM) (NOVA nanoSEM, FEI Japan). A drop of optimized niosomal formulation NS_3_ was mounted on aluminum stub with adhesive silver tape. The stubs were kept overnight under vacuum and then sputter-coated with gold.

### 2.5. Evaluation of niosomal gel

#### 2.5.1. Determination of pH

The pH of the niosomal gel was determined using digital pH meter (WTW pH197i, Germany). Before measurement, pH meter was calibrated and readings were taken by dipping the glass electrode into niosomal gel and comparing with commercial fusidic acid product (Fusidin) [25].

#### 2.5.2. Rheological studies

Niosomal gel of fusidic acid (NG1) stored at different temperatures, i.e 8°C and 25°C, was evaluated for rheological parameters, i.e., shear stress, shear rate, and viscosity. The study was conducted by spindle rheometer (DV-III Ultra Rheometer, Brookfield Engineering Laboratories, Inc). Approximately 0.5 g of niosomal gel (NG1) stored at different temperature was applied on the plate. The software program RheocalcT was used as supporting system. Data was taken at different time intervals for total period of 28 days. Viscosity measurements was made by applying power law, as

(2) $$\tau =K\gamma^{n}$$

where τ = shear stress, γ = shear rate, K = consistency index, n = flow index.

#### 2.5.3. Spreadability studies

The spreadability of the niosomal gel NG1 was determined using the parallel plate method [25]. In this method, 0.5 g of the gel was placed within a circle of 1 cm diameter (premarked on a glass plate), over which a second glass plate was placed. A weight of 500 g was allowed to rest on the upper glass plate for 5 minutes. The increase in the diameter due to gel spreading was noted.

#### 2.5.4. Ex vivo *permeation studies*

*Ex vivo* studies of drug-loaded niosomes-based gel NS_3_G and drug-loaded plain gel were conducted by using Franz diffusion cell. Receptor volume capacity was 12 ml, and the surface area was 1.76 cm^2^. The excised skin of albino rat was used as a model for diffusion. Skin was placed between the donor and the receptor compartment of flow through cells. Phosphate buffer solution with the pH value of 7.4 was filled in the receptor section, and its role was to act as a simulated blood medium. The apparatus was set at 300 rpm stirring speed and 37°C temperatures. The formulation was spread evenly on the membrane. Sample of 2 mL was withdrawn at periodic intervals for 12 hours, and same volume of buffer solution was added to the cell for maintaining sink conditions. Concentration of drug in each sample was measured by UV-visible spectrophotometer [26]. The graph was drawn between cumulative amount of drug permeated and time for both formulation and control. Flux of drug was calculated across the skin by using the following equation

(3) F=KpxCi

F = transdermal flux, Kp = Co-efficient of apparent permeability, Ci = Initial concentration of drug.

## 3. Results and discussion

### 3.1. Preparation of niosomes and niosomes-based gel

Niosomes containing fusidic acid were successfully prepared using thin film hydration technique. Sorbitan monostearate (Span 60) and polyoxyethylene sorbitan monostearate (Tween 60) have been evaluated for their ability to form vesicles with different concentrations of cholesterol (50% to 30% mol/mol). Table 1 showed varying ratios of surfactants and cholesterol. Since cholesterol has been known to stabilize vesicles, influence of cholesterol addition to surfactant system on niosomes formation was also investigated. Hexadecyl pyridinium chloride was incorporated (4.25 mg) into niosomes to prevent aggregation and coalescence of vesicles to maintain their integrity and uniformity [23]. Hexadecyl pyridinium chloride provides sufficient stability to niosomes reported earlier [27]. The hydrophobic parts of the surfactants are shielded from aqueous environment, and the hydrophilic head groups contact with water to obtain closed bilayer structure. Based on the characteristics, niosomal formulation NS3 was screened out as optimum one and converted into deliverable drug delivery system, i.e gel. Carbopol 934 was utilized for preparing gel from niosomal formulations.

### 3.2. Fourier transform infrared spectroscopy (FTIR)

**Figure 1.**
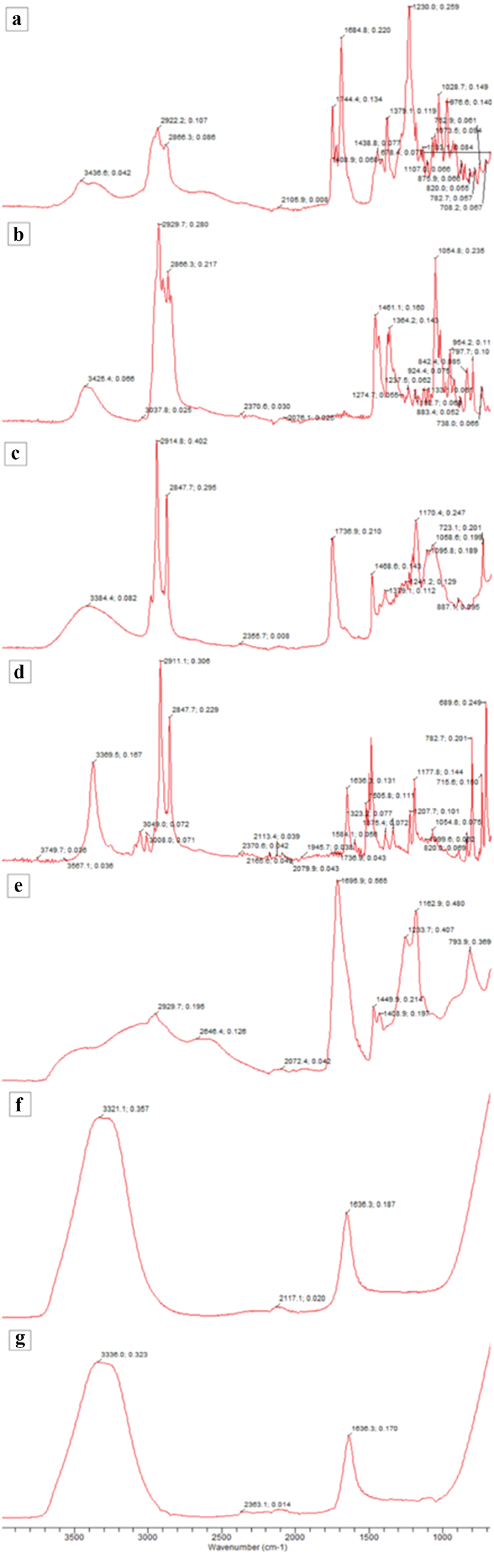
FTIR spectra of fusidic acid (A), cholesterol (B), Span 60 (C), hexadecyl pyridinium chloride (D), Carbopol 934 (E), niosome formulation NS_3_ (F), and niosome formulation NT_3_ (G).

The FTIR results active drug (Fusidic acid), excipients (Cholesterol, Span, Hexadecyl pyridinium chloride, and Carbopol), and Niosomal drug formulation (NS_3_ and NT_3_) are presented in Figure 1. The characteristic absorption peaks of fusidic acid were found at 3436.6 cm^−1^, 3364.04 cm^−1^, 2922.2 cm^−1^, 2622.29 cm^−1^ (carboxylic acid O-H stretching), 1684.08 cm^−1^ (carboxylic acid C = O stretching), 1438.8 cm^−1^, and 1379.75 cm^−1^ (aromatic C = C) peaks at 1230.0 cm^−1^ and 1028.7 cm^−1^, which confirm the aromatic structure of fusidic acid. The spectrum of the Span 60 contained peaks of the hydroxyl group at 3384.40 cm^−1^, strong aromatic –CH3 group at 2914.8 cm^−1^, and the strong C = O ester bond at 1736.9 cm^−1^. For carbopol 934, its FTIR spectra showed a peak in the 3000–2950 cm^−1^ range, representing OH stretching vibration, i.e., ʋ_O-H_ and intramolecular hydrogen bonding. The prominent peak between 1750 and 1700 cm^−1^ was assigned to carbonyl C = O stretching band, i.e., ʋ_C = O_ while the peak at 1450 to 1400 cm^−1^ was for C-O/O-H. The band at 1250 to 1200 cm^−1^ suggested ʋ_C-O-C_ of acrylates. The ethereal crosslinking, indicated by the prominent peak at 1162.9 cm^−1^, represented a stretching vibration of ʋ_C-O-C_ group. The band between 850 and 800 cm^−1^ suggested out of plane bending of C = CH, i.e., bending vibration of aromatic enes [28]. On the other hand, fusidic acid, when incorporated in niosomes, exhibited significant physical interaction as most of the fusidic acid peaks were diffused in the FTIR spectra of niosomal formulations NS_3_ and NT_3_. This might be due to the effect of high temperature during niosomes development, which resulted in the molecular dispersion of drug within the microenvironment of niosomes.

### 3.3. Drug entrapment studies

**Figure 2.**
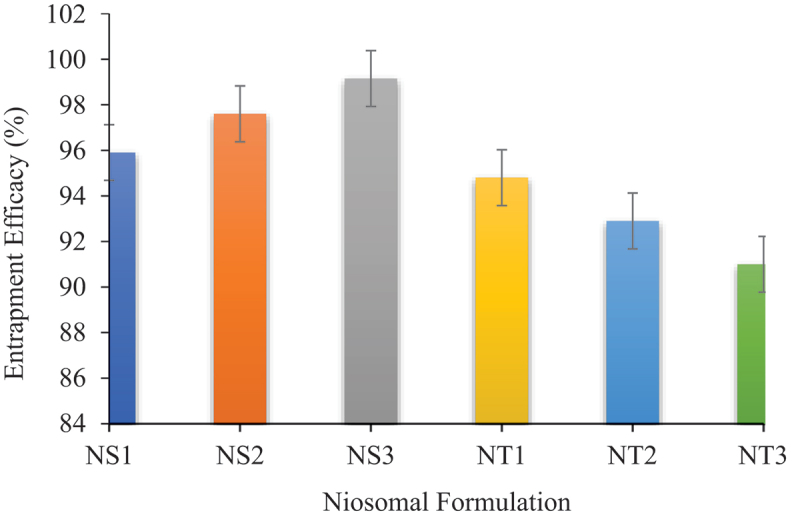
Entrapment efficiency of niosomal formulations.

The success of drug delivery system depends upon the amount of drug trapped by it and minimal wastage of active moiety. The EE values were found to be 95.9–99.15% for the Span 60 formulations and 91–94.8% for Tween 60 formulations (Figure 2). The EE of niosomes developed from Span 60 showed increased values with decrease in cholesterol concentration, whereas it showed decreased values in case of Tween 60 niosomes. Formulation S3 showed the highest EE, and it might be due to the fact that the S3 formulation had the highest surfactant to cholesterol ratio of 7:3 (Table 1), which means it has low cholesterol and high amount of Span 60. The reason for this is that the increased cholesterol may compete with drug to be entrapped into bilayer and decreased skin permeation [29].

The NS3 formulation of fusidic acid niosomes having highest EE was incorporated into Carbopol gel, and this niosomal gel formed was further characterized. Niosomal gel characterization was done by checking their pH, rheology, and spreadability. Its physical appearance was checked by passing it between thumb and finger [30]. The consistency and homogeneity of niosomal gel of fusidic acid was good with no coarse particles. Apparently, it was white in color. The spreadability and rheology of formulation are important parameters as they influence adherence of formulation to the skin [30].

### 3.4. Vesicle size, PDI, and zeta potential of niosomes

Vesicle size of niosomes is very important for the delivery and clearance of drug. The average size of fusidic acid loaded niosomes was found in range from 377.2 to 725.4 nm (Table 2). The size of niosomes depends on surfactant type and cholesterol content in formulation. Moreover, the size of niosomes is also influenced by the hydrophilic–lipophilic balance (HLB) values of surfactants [31]. HLB is a dimensionless parameter for surfactants, which is known as a time-saving guide to surfactant selection [32]. It is very useful tool for controlling entrapment efficiency and size of niosomal systems. HLB ranges from 0 to 20 for nonionic amphiphiles, and lower HLB value (<9) indicates lipophilic nature of nonionic surfactant (oil-soluble) and higher HLB (>11) refers to hydrophilic character of surfactant (water-soluble) [33]. Among the nonionic surfactants employed in the present study, Tween 60 has high HLB value, i.e 14.9, and therefore has lower hydrocarbon chain volume in comparison to hydrophilic surface area. Therefore, Tween 60 yielded niosomes with large vesicle size compared to Span 60-based niosomes, which has low HLB value, i.e 4.7 [31]. Moreover, Span 60 has HLB value of 4.7 and higher phase-transition temperature (53–57°C) [34]. Smaller size of niosomes attained with Span 60 is due to its low HLB value, it might be due to surface free energy, which decreases with increasing hydrophobicity [35]. In addition to this, smaller sized niosomes have greater ability to penetrate skin layers as compared to larger sized niosomes [29]. Cholesterol is a main ingredient of niosomes structure, which improves the mechanical strength of vesicles [36]. As two types of nonionic surfactants were employed in this study, the effect of cholesterol was found different for each surfactant system. In case of Span 60-based niosomes, increase in vesicle size was observed with decrease in cholesterol contents (Table 2). It is reported that high cholesterol contents yield smaller sized niosomes due to increased lipophilicity among vesicular layers [37], and our finding were consistent with previous investigations. In addition to this, higher cholesterol concentrations in niosomes increase bilayer hydrophobicity, thus increasing surface energy and particle size reduction [38]. Among Span 60-based niosomes, NS1 formulation showed smallest particle size and NS3 revealed largest vesicle size. On the other hand, Tween 60-based niosomes exhibited different trend for cholesterol effect on size. The size of vesicles was decreased as cholesterol concentration was decreased (Table 2). Tween 60 has a large hydrophilic head group with high HLB (14.9), which cannot form vesicle without cholesterol. Therefore, addition of cholesterol to Tween 60 made the entire critical packing parameters (CPP) value achieve suitable value 0.5–1 for vesicle formation [39]. The results indicated that niosomal size decreased (P < 0.05) linearly with decreasing cholesterol concentration in agreement with previous findings [40]. Among the formulations, NS3 showed comparatively lesser degree of polydispersity (0.245). The charges on niosomes affect the stability of niosomes as well as *in vivo* fate of colloidal carriers. When zeta potential increases, the charged particles repel each other and prevents aggregation [41]. The zeta potential of niosomes of different formulations range from −3.56 mV to −25.2 mV (Table 2). The negative charge was due to the presence of negatively charged hexadecyl pyridinium chloride.

**Table 2.** Average vesicle size, PDI and zeta potential of niosomal formulations (n = 3)

Niosomes code	Vesicle size (d.nm)	PDI	Zeta potential (mV)
NS1	379.8 ± 3.4	0.444 ± 0.56	−18.6 ± 5.7
NS2	426.2 ± 2.2	0.356 ± 0.47	−24.8 ± 3.4
NS3	547.2 ± 3.5	0.245 ± 0.08	−25.2 ± 2.2
NT1	725.4 ± 2.5	0.809 ± 0.23	−10.5 ± 2.7
NT2	650.2 ± 8.4	0.633 ± 0.67	−8.61 ± 4.7
NT3	377.2 ± 4.3	0.671 ± 0.53	−3.56 ± 5.5

Each value represents the mean± SD (n = 3).

### 3.5. Morphology of niosomes

**Figure 3.**
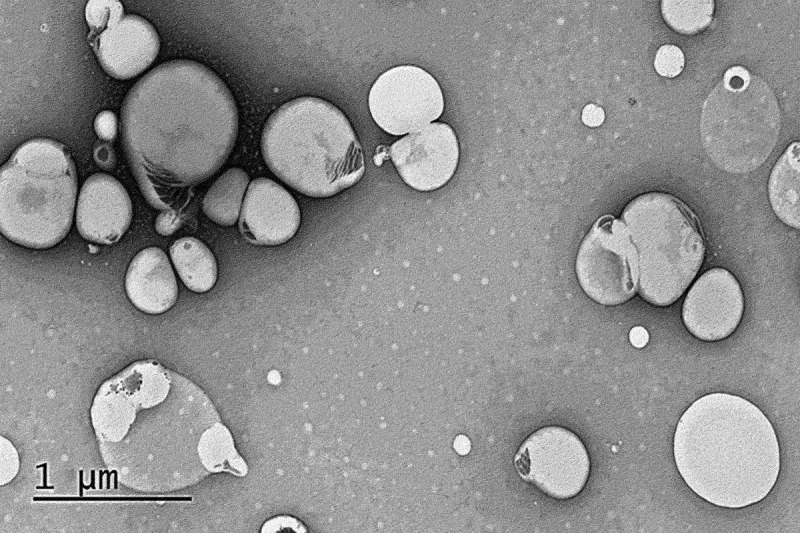
SEM micrograph of optimized niosomal formulation (NS_3_). Photomicrograph was taken on acceleration voltage of 10 kV at diverse amplification.

The SEM micrographs of NS_3_ formulation showed spherical-shaped vesicles with distinct layer containing aqueous core as shown in Figure 3. Furthermore, mean vesicle size was in good agreement with that attained using DLS experiment.

### 3.6. Characterization of niosomesbased gel (NS_3_G)

## 3.6.1. pH determination

**Figure 4.**
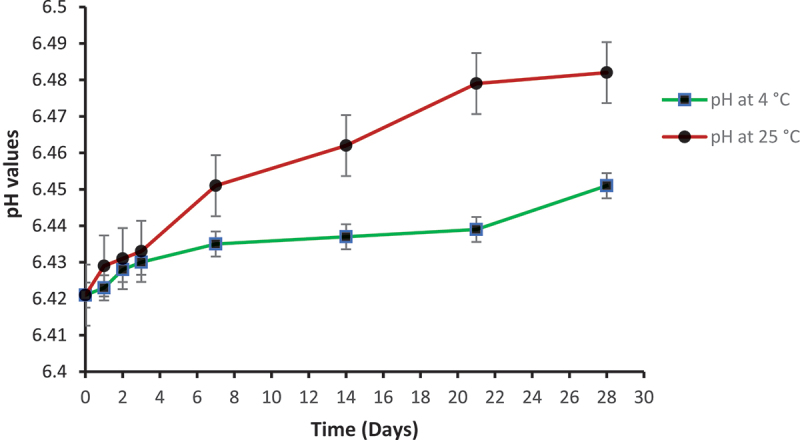
pH values of NS_3_G gel at 4°C and 25°C. Each value represents the mean± SD (n = 3).

In the preparation of topical drug delivery system, pH of the formulation is very important. The little change in pH of topical gel, either basic or acidic, can cause skin irritation upon application [42]. pH of niosomal gel NS_3_G was measured at 4°C and 25°C using pH meter. The pH at both temperatures was found within the range of 6.421–6.482 (Figure 4). These results clearly indicate that fusidic acid niosomal gel is suitable for topical use as its pH is close to normal skin pH.

### 3.7. Rheological studies

**Figure 5.**
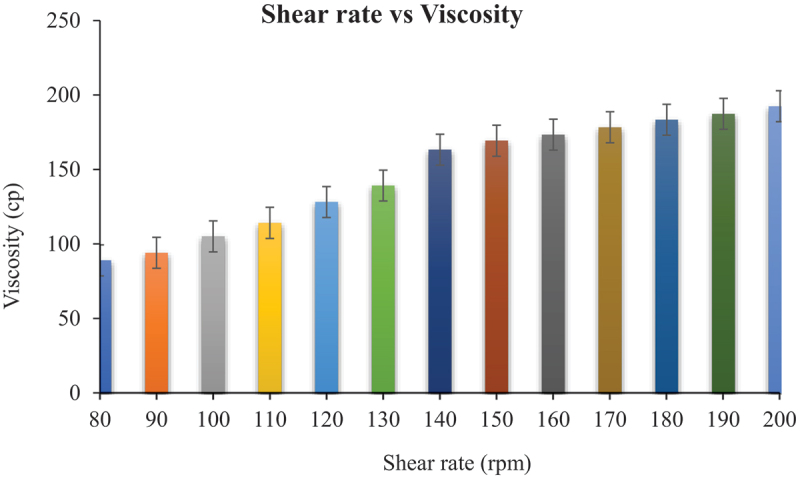
Rheogram of niosomes-based gel (NS_3_G) containing fusidic acid. Each value represents the mean± SD (n = 3).

The results of rheology are presented in Figure 5, as rheology was performed to check the flow behavior of formulations. It determines the packaging characteristics and adherence of dosage form on the site of application. The rheological behavior of dispersion of vesicles depends on the interaction between vesicles and the vesicles deformability [43].

### 3.8. Spreadability studies

Spreadability is an important characteristic of gel; it showed the behavior of the gel applied on the skin. Spreadability is the term expressed to denote the extent of area to which the gel readily spreads on application to the skin [44]. Spreadability is of pivotal significance for topical drug delivery systems from patient compliance point of view [45]. The spreadability is an essential requirement for consistent and ease of application of the topical gel [46]. Spreadability also affects the therapeutic efficacy of the drug. It facilitates smooth application of gel on to the skin and enhances the patient acceptance [47]. The spreadability of NS3G is shown in Table 3 at different temperatures and different time. The results showed good spreadability of topical gel.

**Table 3.** Spreadability of fusidic acid niosomal gel

Time	Spreadability values (g.cm/s)	
	At 8°C	At 25°C
0 day	3.7 ± 1.4	3.7 ± 3.4
1 day	3.7 ± 2.3	3.7 ± 2.5
2 days	3.7 °± 3.2	3.7 ± 1.5
3 days	3.6 ± 1.1	3.6 ± 2.2
7 days	3.6 ± 2.5	3.6 ± 1.4
14 days	3.6 ± 3.3	3.6 ± 2.6
21 days	3.5 ± 1.5	3.5 ± 1.2
28 days	3.5 ± 3.2	3.5 ± 2.4

The results are presented as mean ± SD, n = 3.

### 3.9. Ex vivo *permeation studies*

**Figure 6.**
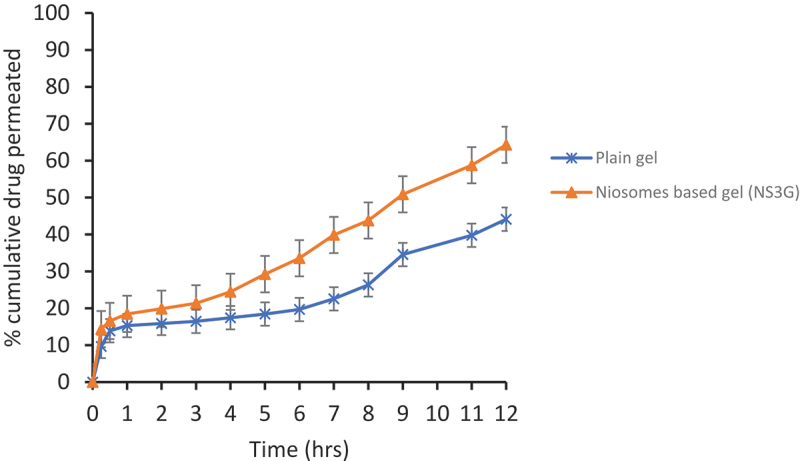
Ex vivo permeation profile of plain gel (Control) and niosomal gel (NS_3_G). Each value represents the mean± SD (n = 3).

The *ex vivo* permeation studies of niosomal gel were also performed to check the skin permeation of fusidic acid when entrapped in niosomes. This study was conducted by using Franz Diffusion Cell. The niosomal gel of fusidic acid has shown greater skin permeation as compared to control gel. The reason is that the drug when entrapped in niosomes helps pass the drug through hydrophilic dermal region easily resulting in increased skin permeation [48]. In Figure 6, results show the percentage cumulative drug permeated versus time of niosomal gel (NS_3_G) and control gel. The value of permeation was high for niosomal gel across the skin than the control gel. The flux value of developed formulation is 80.02 ug/cm^2^/hr, which is greater than the control gel whose value was 15.98 ug/cm^2^/hr; however, co-efficient of permeability for drug formulation is 0.036694 and for control it was 0.007266. Finally, enhancement ratio of niosomal gel comes out to be 5.050.

## Conclusion

Span 60- and Tween 60-based niosomes were successfully prepared using thin film hydration method. The investigated ratios of surfactant to cholesterol (5:5, 7:4, 6:4) produced niosomes. Among all formulas, NS_3_ niosomal formulation was considered as optimized because of maximum entrapment efficiency of fusidic acid achieved. Niosomes were of colloidal size range with less PDI values, indicating homogenous nature of formulations. The selected niosome formulation (NS3) was incorporated into carbopol gela and evaluated for pH, spreadability, rheological behavior, and permeation profile. The permeability parameters including flux and coefficient of permeability were increased 5.05 times with niosomes-based gel compared to plain gel. The results concluded that niosomes-based gel of fusidic acid can act as alternative dosage form for topical treatment of different skin infections.
